# Evaluation of Cytotoxicity, Genotoxicity and Hematotoxicity of the Recombinant Spore-Crystal Complexes Cry1Ia, Cry10Aa and Cry1Ba6 from *Bacillus thuringiensis* in Swiss Mice

**DOI:** 10.3390/toxins6102872

**Published:** 2014-09-29

**Authors:** Ingrid de Souza Freire, Ana Luisa Miranda-Vilela, Lilian Carla Pereira Barbosa, Erica Soares Martins, Rose Gomes Monnerat, Cesar Koppe Grisolia

**Affiliations:** 1 Department of Genetics and Morphology, Institute of Biological Sciences, University of Brasilia, Brasília 70910-900, Brazil; E-Mails: ingridfgen@gmail.com (I.S.F.); analuisamv@uol.com.br (A.L.M.-V.); liliancpbarbosa@hormail.com (L.C.P.B.); grisolia@unb.br (C.K.G.); 2 Faculty of Medicine, Faciplac, Campus Gama 72460-000, Brazil; 3 Instituto Mato-Grossense do Algodão–IMAmt/Faculdades Integradas ICESP/Promove de Brasília, Brasília 78008-000, Brazil; E-Mail: es.martins2@gmail.com; 4 Laboratory of Bacteriology, Centro Nacional de Recursos Genéticos (CENARGEN), Brasília 70770-917, Brazil; E-Mail: rose.monnerat@embrapa.br

**Keywords:** biopesticides, spore-crystal, micronucleus, endotoxins

## Abstract

The insecticidal properties of Cry-endotoxins from *Bacillus thuringiensis* (Bt) have long been used as spore-crystals in commercial spray formulations for insect control. Recently, some Bt-endotoxin genes have been cloned in many different plants. Toxicological evaluations of three spore-crystal endotoxins, BtCry1Ia, BtCry10Aa and BtCry1Ba6 from *B. thuringiensis*, were carried out on mice to understand their adverse effects on hematological systems and on genetic material. These three spore-crystals have shown toxic activity to the boll weevil, which is one of the most aggressive pests of the cotton crop. Cry1Ia, Cry10Aa and Cry1Ba6 did not increase the micronucleus frequency in the peripheral erythrocytes of mice and did not cause changes in the frequency of polychromatic erythrocytes. However, some hematologic disburbances were observed, specifically related to Cry1Ia and Cry1Ba6, respectively, for the erythroid and lymphoid lineage. Thus, although the profile of such adverse side effects can be related to their high level of exposure, which is not commonly found in the environment, results showed that these Bt spore-crystals were not harmless to mice, indicating that each spore-crystal endotoxin presents a characteristic profile of toxicity and might be investigated individually.

## 1. Introduction

*Bacillus thuringiensis* (Bt) is an important biological control agent, widely used in agriculture around the world due to its larvicidal activity. These effects are caused by crystal proteins (endotoxins) released during the sporulation phase of the bacteria. Due to its well-known insecticidal activity, many Bt-based commercial formulations are available on the market [1].

The endotoxins are released as protoxins by the bacteria and, after being ingested by the larvae of the insect, are cleaved in the alkaline pH of its midgut, becoming toxic [2]. These fragments bind to cell receptors in the midgut of the larvae, causing pores in the membrane and consequently cell death [3]. In a few days, the larvae gut is broken down causing larvae death by septicemia or inanition [4,5]. Because of its specificity to target organisms, it is believed that the effects of Bt on the environment are minimal [6]. However, the capacity of Bt spore-crystals to replicate and disperse throughout an ecosystem have caused concern, mainly due to their extensive use in biological control and now in genetically modified plants. Therefore, it is important to evaluate potential interactions of Bt with non-target organisms [6]. Despite widespread adoption of genetically modified crops based on Bt genes by many countries, controversies about their advantages and disadvantages still remain. Civil society groups tend to emphasize potential risks of this kind in GM crops, raising questions on risks to the environment and human health [7].

The cotton industry is one of the biggest agribusinesses, generating income and products for the world market. However, cotton crops are vulnerable to many pests; in Brazil, the cotton boll weevil, *Anthonomus grandis* Boheman (Coleoptera: Tenebrionidae), is the most important cotton pest, and is considered one of the most aggressive due to the damage that it causes to the crop and because of difficulties in controlling it [8]. *A. grandis* also used to be the most important insect-pest on cotton in North America. Due to the Boll Weevil Eradication Program, sponsored by the USDA, an integrated pest management strategy has been successful in controlling boll weevil populations. In South America, nevertheless, boll weevil populations are still causing great damage to cotton crops, destroying cotton plant floral buds and bolls. Due to their high reproductive rate in tropical areas and to the endophytic behavior of earlier developmental stages, infestation levels increase fast and, unless control measures are adopted, damage can lead to total loss of production. The ineffectiveness and harmful aspects of using chemical control to arrest the infestation have led to a search for more efficient control strategies, of which the most promising are in the biotechnological area. Genetically modified (GM) crops to control insect pests are now widely used. Several proteins have been introduced in plants in order to control insects, mainly the *Bacillus thuringiensis* toxins [9].

Chemical control is the most common approach in place to control boll weevil infestations in cotton, but pesticide use requires large investments, which may affect the profitability of this crop. Biological control agents based on *Bacillus thuringiensis* are an economical alternative to chemical insecticides and are environmentally friendly [8]. Bt toxins have been used for many years to control insects and more recently many of the genes of this bacterium have been inserted into plants, which start to produce the toxin. The toxins used in this study showed high toxicity to the cotton boll weevil, which is one of the most important cotton pests and for which there is no effective way to control. It is essential that the adverse effects that may be caused by these toxins are known, and this study has attempted to contribute to this purpose.

These three new crystal-proteins, BtCry1Ia [10], BtCry10Aa [11] and BtCry1Ba6 [12], showed efficacy against the boll weevil *Anthonomus grandis* Boheman (Coleoptera: Tenebrionidae). Furthermore, these endotoxins have demonstrated potential for use in the biological control of insects in cotton crops as well as for cloning in cotton plants, becoming genetically resistant. Brazilian researchers are currently searching for new biopesticides against the cotton boll weevil. Furthermore, insect-resistance to conventional organophosphorus insectides is a long-standing problem in agriculture. The literature has also reported insect-resistance to Bt cry-endotoxins [13]. Therefore, screening for new cry-endotoxins that are toxic to *Anthonomus grandis* has been carried out, and Cry1Ia, Cry10Aa and Cry1Ba6 have shown efficacy as an insecticide. The aim of this study was therefore to evaluate the adverse effects of Bt spore-crystals, expressing Cry1Ia, Cry10Aa and Cry1Ba6 in Swiss mice using different endpoints, such as micronucleus test from peripheral erythrocyte cells, cytotoxicity through inhibition of polychromatic erythrocyte cells proliferation and hematotoxicity analyzing variations in the hemogram, leukogram and plateletgram.

## 2. Results and Discussion

Table 1 shows results of the mutagenicity test. Almost all endotoxins tested did not increase the levels of micronuclei, compared with the control group (*p* > 0.05), except for Cry10Aa (1 × 10^9^ spores/kg). The relationship between percentage of polychromatic erythrocytes (%PCE) and percentage of normochromatic erythrocytes (%NCE) was not statistically changed in any treatment group in relation to the control group, which means no adverse effects on the proliferation of the bone marrow tissue.

In respect to the Erythrogram (Table 2), although no significant differences were observed for the erythrocyte number, hemoglobin (HGB) and hematocrit (HCT) compared to control, all treated groups presented a significant reduction in values of the mean corpuscular hemoglobin (MCH) (*p* < 0.01), although all of them were inside the reference intervals described for mice [14]. Except for Cry1Ba6 (4 × 10^8^ spores/kg), the treatments also reduced the mean corpuscular hemoglobin concentration (MCHC), but also inside the reference intervals for mice [14]. Cry10Aa (5 × 10^9^ spores/kg), Cry10Aa (1 × 10^10^ spores/kg), and all Cry1Ba6 treatments also significantly reduced the mean corpuscular volume (MCV) values, while Cry1Ia (4 × 10^9^ spores/kg) increased the red cell distribution width (RDW) above the reference values (*p* < 0.01) [15].

In the analysis of the leukogram, almost all parameters showed no significant statistical differences compared to the control, except Cry10Aa (1 × 10^10^ spores/kg), which significantly reduced the lymphocyte percentage; Cry1Ba6 (4 × 10^8^ spores/kg) and Cry1Ba6 (2 × 10^9^ spores/kg), which significantly decreased the number of neutrophils + monocytes; and Cry1Ba6 (4 × 10^9^ spores/kg), which significantly reduced both (Table 3).

**Table 1:** Results of micronucleus test carried out in polychromatic and normochromatic peripheral erythrocytes of mice. Percentages of polychromatic erythrocytes were used to estimate inhibition of cell proliferation.

Groups	Exposures	PCE	NCE	MN-PCE	MN-NCE	%PCE	%NCE
1	Control (Filtered water)	564.17 ± 31.02	2435.83 ± 31.02	0.67 ± 0.33	10.67 ± 1.54	18.81 ± 1.03	81.19 ± 1.03
2	Cry10Aa (1 × 10^9^ spores/kg)	267.33 ± 37.52 *	2732.67 ± 37.52 *	0.17 ± 0.17	9.67 ± 2.04	8.91 ± 1.25 *	91.09 ± 1.25 *
3	Cry10Aa (5 × 10^9^ spores/kg)	549.50 ± 66.56	2450.50 ± 66.56	0.67 ± 0.21	9.33 ± 1.17	18.32 ± 2.22	81.68 ± 2.22
4	Cry10Aa (1 × 10^10^ spores/kg)	505.83 ± 65.7	2494.17 ± 65.70	0.17 ± 0.17	7.83 ± 1.58	16.86 ± 2.19	83.14 ± 2.19
5	Cry1Ba6 (4 × 10^8^ spores/kg)	471.50 ± 44.31	2528.50 ± 44.31	0.33 ± 0.21	15.50 ± 3.76	15.72 ± 1.48	84.28 ± 1.48
6	Cry1Ba6 (2 × 10^9^ spores/kg)	565.17 ± 80.97	2434.83 ± 80.97	0.17 ± 0.17	17.33 ± 3.66	18.84 ± 2.70	81.16 ± 2.70
7	Cry1Ba6 (4 × 10^9^ spores/kg)	503.50 ± 60.47	2496.50 ± 60.47	0.33 ± 0.21	12.17 ± 3.25	16.78 ± 2.02	83.22 ± 2.02
8	Cry1Ia (4 × 10^8^ spores/kg)	308.33 ± 38.87	2691.67 ± 38.87	0.67 ± 0.33	8.33 ± 1.45	10.28 ± 1.30	89.72 ± 1.3
9	Cry1Ia (2 × 10^9^ spores/kg)	454.33 ± 71.11	2545.67 ± 71.11	0.83 ± 0.40	11.33 ± 1.43	15.14 ± 2.37	84.86 ± 2.37
10	Cry1Ia (4 × 10^9^ spores/kg)	520.00 ± 44.59	2480.00 ± 44.59	0.67 ± 0.33	11.67 ± 2.78	17.33 ± 1.49	82.67 ± 1.49

Notes: PCE = polychromatic erythrocyte; NCE = normochromatic erythrocyte; MN = frequency of micronucleus in 3000 cells analyzed. The data correspond to the means and to the standard error of mean (SEM); Asterisks indicate significant differences (*p* < 0.05) compared to control, detected by the Tukey test in the 2-to-2 comparisons.

**Table 2:** Results of the Erythrogram from whole blood of Swiss mice after 72 h of exposure to three Bt-endotoxins showing the main blood parameters analyzed.

Group	Exposures	Erythrocytes (×10^6^/μL)	HGB (g/dL)	HCT (%)	MCH (pg)	MCHC (g/dL)	MCV (fL)	RDW (%)
1	Control (filtered water)	7.72 ± 0.20	12.22 ± 0.21	29.85 ± 0.61	15.85 ± 0.23	40.95 ± 0.38	38.68 ± 0.33	14.68 ± 0.89
2	Cry10Aa (1 × 10^9^ spores/kg)	8.20 ± 0.16	11.97 ± 0.20	30.48 ± 0.56	14.60 ± 0.14 *	39.27 ± 0.14 *	37.17 ± 0.38	16.20 ± 0.36
3	Cry10Aa (5 × 10^9^ spores/kg)	7.40 ± 0.21	10.63 ± 0.35	26.95 ± 0.88	14.37 ± 0.13 *	39.45 ± 0.10 *	36.38 ± 0.27 *	15.95 ± 0.29
4	Cry10Aa (1 × 10^10^ spores/kg)	7.55 ± 0.33	10.85 ± 0.53	27.60 ± 1.35	14.33 ± 0.23 *	39.27 ± 0.29 *	36.52 ± 0.59 *	16.70 ± 0.68
5	Cry1Ba6 (4 × 10^8^ spores/kg)	8.32 ± 0.12	12.15 ± 0.18	30.22 ± 0.38	14.60 ± 0.20 *	40.23 ± 0.25	36.35 ± 0.42 *	15.40 ± 0.46
6	Cry1Ba6 (2 × 10^9^ spores/kg)	8.46 ± 0.23	11.97 ± 0.33	30.58 ± 0.83	14.15 ± 0.11 *	39.12 ± 0.14 *	36.13 ± 0.22 *	16.53 ± 0.41
7	Cry1Ba6 (4 × 10^9^ spores/kg)	8.73 ± 0.18	12.30 ± 0.39	31.53 ± 0.76	14.08 ± 0.19 *	38.98 ± 0.33 *	36.13 ± 0.36 *	16.50 ± 0.72
8	Cry1Ia (4 × 10^8^ spores/kg)	7.72 ± 0.12	11.42 ± 0.18	28.68 ± 0.42	14.80 ± 0.08 *	39.82 ± 0.31 *	37.22 ± 0.36	17.55 ± 0.48
9	Cry1Ia (2 × 10^9^ spores/kg)	8.04 ± 0.14	11.73 ± 0.24	29.70 ± 0.39	14.62 ± 0.21 *	39.48 ± 0.32 *	37.00 ± 0.42	17.82 ± 0.69
10	Cry1Ia (4 × 10^9^ spores/kg)	8.11 ± 0.37	11.63 ± 0.53	30.17 ± 1.22	14.43 ± 0.12 *	38.50 ± 0.27 *	37.25 ± 0.33	19.02 ± 0.83 *

Notes: The data correspond to the means and to the standard error of mean (SEM). RBC = Red Blood Cells; HGB = Hemoglobin; HCT = Hematocrit; MCV = Mean Corpuscular volume; MCH = Mean Corpuscular hemoglobin; MCHC = Mean corpuscular hemoglobin concentration; RDW = Red cell distribution width (represents an indication of the amount of variation in cell size or anisocytosis); g/dL = grams per deciliter; fl = fentoliters; pg = picograms. Asterisks indicate significant differences (*p* < 0.01) compared to control, detected by the Mann Whitney U test (MCHC) or the Tukey test (other variables) in the 2-to-2 comparisons.

**Table 3:** Results of leukogram of Swiss mice obtained from whole blood after 72 h of exposure to three Bt-endotoxins showing the main leukocytary parameters analyzed.

Groups	Exposures	Total of leukocytes (%)	Lymphocytes (%)	Neutrophils + Monocytes (%)	Eosinophils (%)	Lymphocytes (×10^3^/μL)	Neutrophils + Monocytes (×10^3^/μL)	Eosinophils (×10^3^/μL)
1	Control (Filtered water)	9.67 ± 1.19	51.32 ± 3.19	46.25 ± 3.07	2.43 ± 1.64	4.92 ± 0.63	4.48 ± 0.66	0.27 ± 0.19
2	Cry10Aa (1 × 10^9^ spores/kg)	8.18 ± 1.16	47.33 ± 4.69	51.65 ± 4.85	1.02 ± 0.45	3.93 ± 0.73	4.15 ± 0.61	0.10 ± 0.07
3	Cry10Aa (5 × 10^9^ spores/kg)	6.50 ± 1.07	43.27 ± 6.24	54.87 ± 6.22	1.87 ± 0.38	2.98 ± 0.76	3.42 ± 0.56	0.10 ± 0.03
4	Cry10Aa (1 × 10^10^ spores/kg)	9.85 ± 3.52	35.20 ± 4.63 *	61.08 ± 6.43	3.72 ± 2.25	2.87 ± 0.72	6.73 ± 2.80	0.25 ± 0.11
5	Cry1Ba6 (4 × 10^8^ spores/kg)	6.12 ± 1.20	59.63 ± 3.80	38.35 ± 3.25	2.02 ± 1.92	3.75 ± 0.81	2.35 ± 0.48 *	0.02 ± 0.02
6	Cry1Ba6 (2 × 10^9^ spores/kg)	5.28 ± 1.00	61.87 ± 4.26	35.82 ± 4.13	2.32 ± 1.90	3.47 ± 0.74	1.73 ± 0.34 *	0.08 ± 0.07
7	Cry1Ba6 (4 × 10^9^ spores/kg)	5.73 ± 1.47	65.02 ± 2.95 *	34.05 ± 2.58	0.93 ± 0.55	3.90 ± 1.15	1.80 ± 0.35 *	0.03 ± 0.02
8	Cry1Ia (4 × 10^8^ spores/kg)	8.50 ± 0.88	52.58 ± 3.65	46.75 ± 3.74	0.67 ± 0.32	4.58 ± 0.67	3.87 ± 0.32	0.05 ± 0.02
9	Cry1Ia (2 × 10^9^ spores/kg)	9.83 ± 0.94	57.37 ± 5.82	41.73 ± 5.47	0.90 ± 0.53	5.73 ± 0.91	4.02 ± 0.50	0.08 ± 0.03
10	Cry1Ia (4 × 10^9^ spores/kg)	8.52 ± 1.40	44.37 ± 4.73	53.97 ± 4.76	1.67 ± 0.46	3.82 ± 0.76	4.57 ± 0.86	0.13 ± 0.06

Notes: The data correspond to the means and to the standard error of mean (SEM). Data were generated by Mann Whitney U test. Asterisks indicate significant differences (*p* < 0.05) compared to control, detected by the Mann Whitney U test in the 2-to-2 comparisons.

For the plateletgram, although there were no significant differences in the platelet count (PLT), values of mean platelet volume (MPV) and platelet large cell ratio (P-LCR) significantly increased in almost all treatments, while Cry10Aa (1 × 10^9^ spores/kg) and all Cry1Ia treatments promoted a significantly augmented platelet distribution width (PDW) (Table 4).

**Table 4:** Results show the main values of plateletgram of Swiss mice after 72 h of exposure to three Bt-endotoxins, at three different exposure levels.

Groups	Exposures	PLT (×10^3^/μL)	MPV (fl)	P-LCR (%)	PDW (fl)
1	Control (Filtered water)	1413.33 ± 138.85	5.38 ± 1.08	5.83 ± 1.55	5.60 ± 1.12
2	Cry10Aa (1 × 10^9^ spores/kg)	1149.50 ± 87.72	7.10 ± 0.17 *	11.93 ± 1.34 *	7.02 ± 0.09 *
3	Cry10Aa (5 × 10^9^ spores/kg)	1128.83 ± 64.71	7.07 ± 0.17 *	11.15 ± 1.25 *	7.07 ± 0.15
4	Cry10Aa (1 × 10^10^ spores/kg)	1467.50 ± 206.34	7.13 ± 0.25 *	11.93 ± 1.50 *	6.93 ± 0.26
5	Cry1Ba6 (4 × 10^8^ spores/kg)	1530.00 ± 39.61	6.58 ± 0.03	8.43 ± 0.38	6.68 ± 0.07
6	Cry1Ba6 (2 × 10^9^ spores/kg)	1449.40 ± 90.90	7.14 ± 0.12 *	12.16 ± 0.47 *	7.10 ± 0.17
7	Cry1Ba6 (4 × 10^9^ spores/kg)	1541.33 ± 59.24	7.04 ± 0.25 *	11.76 ± 1.45 *	7.06 ± 0.17
8	Cry1Ia (4 × 10^8^ spores/kg)	1675.00 ± 55.71	6.90 ± 0.09	9.10 ± 0.60	7.13 ± 0.07 *
9	Cry1Ia (2 × 10^9^ spores/kg)	1789.67 ± 180.68	7.00 ± 0.13 *	9.87 ± 0.72	7.20 ± 0.12 *
10	Cry1Ia (4 × 10^9^ spores/kg)	1778.40 ± 200.69	7.34 ± 0.20 *	12.70 ± 1.05 *	7.38 ± 0.21 *

Notes: PLT = platelet count; MPV = mean platelet volume; P-LCR = platelet large cell ratio; PDW = platelet distribution width; fl = fentoliters. Asterisks indicate significant differences compared to the control, detected by the Mann Whitney U test (P-LCR) or the Tukey test (other variables) in the 2-to-2 comparisons (* *p* < 0.05).

Some bio-safety tests have demonstrated that exposures to spore-crystals cause low toxic effects to vertebrates. Laboratory studies have demonstrated that Bt products are non-infectious and are toxic to mammals only at doses over 10^8^ colony forming units (CFU) per mouse and 10^11^ CFU per human. No evidence of illness was found in rats and sheep that were fed on Bt products. Regarding handling pesticides, a “no-risk situation” does not exist. Human inhalation of Bt spores due to spray campaigns can elicit some allergenic process [16]. The Cry proteins are regarded as harmless or nontoxic to mammals, including humans, probably due to the acidified gut, where they break down like many other proteins [17]. However, the toxicity of such Bt spore-crystals to mammals is still an open question regarding mode of action, and human risks cannot be discounted due to the wide use of Bt as a biopesticide as well as in genetically modified crops.

Aquatic exposure of spore-crystals Cry1Aa, Cry1Ab, Cry1Ac e Cry2Aa to zebrafish at different exposure levels showed low toxicity and no genotoxicity, except for Cry1Aa which increased the micronucleus levels in peripheral erythrocyte cells [18]. In addition, in our previous study of *Oreochromis niloticus* exposed to Bt spore-crystals Cry1Ia, Cry10Aa and CyrBa6 for 96 h no mutagenicity was observed through micronucleus test or DNA damage by the comet assay in peripheral erythrocyte cells [19]. Additionally, mice orally treated with *Bt* spore-crystals genetically modified to express individually Cry1Aa, Cry1Ab, Cry1Ac or Cry2A showed no genotoxic effect [20], corroborating our results. Micronuclei may be alternatively detected in circulating normochromatic erythrocytes (NCE), because unlike the spleen of rat and man, the mouse spleen does not remove micronucleated erythrocytes from circulating blood [21].

The micronucleus methodology was chosen rather than the classic test-system in bone marrow cells because the exposures were carried out for 72 h. Furthermore, slides stained by acridine orange distinguish polychromatic erythrocytes from normochromatic erythrocytes in the peripheral blood. Only Cry10Aa, at lower exposure (1 × 10^9^ spores/kg), decreased the frequencies of polychromatic erythrocytes and no dose-effect relationship was observed.

However, some solubilized δ-endotoxins have shown toxicity to mice as well as cytolytic activity to human erythrocytes [22]. Results of *in vitro* hemolysis in cell lines of rat, mouse, sheep, horse, and human erythrocytes suggest that the plasma membrane of erythrocytes may also be the primary target for these toxins [23]. Another study showed that Cry1Ac protein binds to the mucosa surface of the mouse small intestine. The confocal images showed that this protoxin binds mainly to the apical surface and brush border and can induce immune response considering both the oral and intraperitoneal route [24].

In the present study, Bt spore-crystals, Cry1Ia (2 × 10^9^ spores/kg) and Cry1Ia (4 × 10^9^ spores/kg) showed toxicity for the erythrocytes, compatible with a possible hemolysis. This is because, besides significantly reducing MCH and MCHC (*p* < 0.05), albeit inside the reference values for mice [14], the RDW also increased above the reference values [15], which represents an indication of the amount of variation in cell size or anisocytosis. Although in mice anisocytosis is more pronounced than in man, due to higher concentration of reticulocytes, usually from 1% to 6% of circulating erythrocytes, an increase in the percentage of reticulocytes and, consequently in RDW, may be indicative of bleeding or hemolytic anemia [14,15]. Results of plateletgram reinforce this suggestion, since Cry1Ia (2 × 10^9^ spores/kg) and Cry1Ia (4 × 10^9^ spores/kg) also promoted significantly increased MPV, P-LCR and PDW, which can reflect platelet activation and reactivity [25,26,27].

Normally, lower levels of neutrophils/monocytes are related to some bacterial infections, allergic disorders, autoimmune diseases, and treatment with certain medicines, where neutrophils destroy themselves. In this case, exposures to spore-crystal Cry1Ba6 could be related to a response to the infection [28]. On the other hand, in mice, processes or substances that damage hematopoietic stem cells or bone marrow stromal cells are generally first recognized by decreases in neutrophils, monocytes and eosinophils, which have a shorter half-life time, followed by decreases in platelet counts and then in red blood counts [15]. In this context, Cry1Ba6 deserves to be further investigated.

When analyzing hematologic data from mice, not only concurrent controls should be used as the primary comparison for interpretation of treatment-related changes, but also comparisons with reference intervals, which are desirable to put hematologic changes in perspective [15]. Thus, this study has demonstrated that the levels of toxicity depend on the strain of spore-crystal and its exposure levels, as we have also shown in our previous study with these same spore-crystals [19]. Considering that more than 50 Cry proteins have already been well-characterized, and that their structure shows three domains responsible for forming pores in the membranes of the susceptible cells, causing lysis, variations in these domains could be responsible for the variations in toxicity because of their requirement to bind to specific receptors for activity [29].

Furthermore, it might be taken into account that Cry1Ab toxin has already been detected in the blood of non-pregnant women, pregnant women and their fetuses, making such studies particularly relevant [30]. In contrast, no differences were observed in the levels of Bt-metabolites in the urine of rats that were exposed and non-exposed to Bt-rice compared with non-Bt-rice [31]. Another study carried out in our laboratory showed that in some circumstances spore-crystals Cry1Aa (1 × 10^8^ spores/kg) and Cry1Ab (3 × 10^7^ and 10^8^ spores/kg) may affect the hematologic system of mice, causing some disturbances in the white blood cells; however, no genotoxicity after oral exposure was observed [20]. This means that such Bt spore-crystals can reach humans. Rats treated by gavage with 2 × 10^12^ spores/kg body weight, and human volunteers that ingested 3 × 10^9^ spore-crystals for 5 days did not show signs of toxicity [32].

In this context, it is notable that the concentration levels of viable spore-crystals used in this study were much higher than those used in the field. It is well known that Bt spores have shown synergistic activity when associated with Cry proteins, becoming more toxic [33]. In general, the maximum concentration level used in commercial biopesticides based on *B. thuringiensis*, for protection of cotton against Lepidoptera *Alabama argillacea*, is around 6 × 10^11^ viable spores per hectare, whereas in this study the maximum concentration levels used were around 1 × 10^10^ spores/kg [34]. We therefore worked with the worse-case scenario.

## 3. Experimental Section

### 3.1. Bt Spore-Crystals

Three different recombinant spore-crystals in the lyophilized form of BtCry1Ia [10], BtCry1Ba6 [12] and BtCry10Aa [11] were obtained from the Germplasm Bank of the Brazilian Agricultural Research Corporation (EMBRAPA) through its National Genetic Resource and Biotechnology Research Center (CENARGEN), Brasilia/DF, Brazil.

These strains of recombinant spore-crystals were grown in Embrapa medium [35], supplemented with 6 μg mL^−1^ of chloramphenicol [36] for BtCry1Ba6 and BtCry10Aa, with 10 μg mL^−1^ erythromycin [37,38] for BtCry1Ia, incubated for 72 h at 28 °C, and maintained in a shaker. After growing, these were centrifuged at 12,800 × g for 30 min at 4 °C, frozen for 16 h and then lyophilized for 18 h. The colony forming units test (CFU) was carried out to quantify the viable Bt spore-crystals and followed the protocol proposed by Alves and Moraes [39]. Three different concentrations of Bt spore crystals were diluted in saline solution at 0.9% for testing such as: Cry10Aa = 1 × 10^9^ spores/kg, 5 × 10^9^ spores/kg and 1 × 10^10^ spores/kg; Cry1Ba6 = 4 × 10^8^ spores/kg, 2 × 10^9^ spores/kg and 4 × 10^9^ spores/kg; and CryI1a = 4 × 10^8^ spores/kg, 2 × 10^9^ spores/kg and 4 × 10^9^ spores/kg.

### 3.2. Study Design

Swiss albino mice of both genders obtained from the animal facilities of the Faculty of the University of São Paulo (Ribeirão Preto/SP, Brazil) were kept in the animal facility of the Laboratory of Genetics of the University of Brasilia (Brasília, Brazil), housed in plastic cages at room temperature (22 °C ± 2 °C) in a 12 h light/dark cycle with lights on at 6 a.m., and with free access to food and water. For testing we used groups of six male mice aged approximately three months. Mice were orally exposed for 72 h. The test-solutions were previously sonicated [40,41] with three pulses of 1 min in a beacker on ice to maintain the temperature under control and releasing the crystal-proteins to the water to achieve better homogenization. After 72 h of exposure, the animals were anesthetized by an intraperitoneal administration of ketamine (80 mg/kg) plus xylazine (10 mg/kg) and blood samples were collected by cardiac puncture (400 μL) with a heparinized syringe to carry out hemogram [20] for the tests. Animals were euthanized by cervical dislocation according to the American Veterinary Medical Association guidelines for euthanasia [42]. All procedures were approved by the institutional Ethics Committee for Animal Research (Institute of Biological Science, University of Brasília), protocol number 94529/2010. During our experiments, mice were constantly observed, and we did not observe signals of stress or toxicity. Our study design followed classical protocols of the literature, which recommend low and high levels of exposures. In this case high levels of exposures means the simulation of worst-case scenario. It is necessary to know the dose-effect relationship.

### 3.3. Micronucleus and Cytotoxicity Tests

Blood samples were smeared on clean slides, dried at room temperature, and fixed with methanol for 10 min. The smeared preparations were then stained with acridine orange supravital-staining, according to the method of Hayashi *et al*. [43], modified. For each animal, three thousand polychromatic erythrocytes (PCEs) and three thousand normochromatic erythrocytes (NCEs) were examined. However, micronuclei (MN) were scored only in PCEs. PCEs were identified by red fluorescing reticulum in the cytoplasm, and MN fluoresced greenish yellow. NCEs fluoresced as pale green. PCE/NCE relationship was also recorded as a sign of cytotoxicity. Two thousand cells were scored to measure the percentage of PCE among total erythrocytes [44,45]. Slides were examined using a fluorescence Axioskop 2 Zeiss microscope (Carl Zeiss Co., Mainz, Germany).

### 3.4. Hematotoxicity Tests

Hemogram was carried out in a multiple automated hematologic analyzer for veterinary use, Sysmex pocH-100iV Diff (Curitiba, Paraná, Brazil) calibrated for mice, in microtubes containing EDTA as anticoagulant.

### 3.5. Statistical Analysis

Statistical analysis was carried out using SPSS (Statistical Package for the Social Sciences) version 17.0 [46]. Data were expressed as mean ± SEM (standard error of mean), and values of *p* < 0.05 were considered statistically significant. The continuous variables were tested for normal distribution with Shapiro-Wilk. Possible differences among the groups were investigated through ANOVA or Kruskal-Wallis test (when the data were not normally distributed). For significant ANOVA results, Tukey’s post-hoc test was chosen to carry out 2-to-2 comparisons between the treatments. For significant Kruskal-Wallis results, Mann-Whitney U test was performed to verify differences between the treatments (2-to-2 comparisons). The *p*-values with statistical significance (*p* < 0.05) showed here were those compared to the controls.

## 4. Conclusions

In conclusion, the spore-crystals BtCry1Ia, BtCry10Aa and BtCryBa6 did not cause mutagenicity in mice. However, some hematologic disturbances were observed, specifically related to Cry1Ia and Cry1Ba6, respectively, for the erythroid and lymphoid lineage. The profile of such adverse side effects can be related to their high level of exposure, which is not commonly found in the environment. Indeed, results indicated that these Bt spore-crystals were not harmless to mice, indicating that each endotoxin presents a characteristic profile of toxicity and might be investigated individually.
